# 666. Molecular Epidemiology of Methicillin-Resistant *Staphylococcus aureus* in Chile between 1999-2018

**DOI:** 10.1093/ofid/ofab466.863

**Published:** 2021-12-04

**Authors:** Jose R W Martínez, Maria Spencer, Lina M Rivas, Rafael Rios, Lorena Diaz, Lorena Diaz, Jinnethe Reyes, Paul J Planet, Patricia Garcia, Cesar A Arias, Jose Munita

**Affiliations:** 1 Genomics & Resistant Microbes (GeRM), Instituto de Ciencias e Innovación en Medicina, Facultad de Medicina Clínica Alemana, Universidad del Desarrollo, Chile; Millennium Initiative for Collaborative Research on Bacterial Resistance (MICROB-R), Santiago, Region Metropolitana, Chile; 2 Universidad El Bosque, Bogota, Distrito Capital de Bogota, Colombia; 3 Molecular Genetics and Antimicrobial Resistance Unit and International Center for Microbial Genomics, Universidad El Bosque, Bogota, Colombia, Bogota, Distrito Capital de Bogota, Colombia; 4 Children’s Hospital of Philadelphia/UPenn, Philadelphia, Pennsylvania; 5 Pontificia Universidad Catolica de Chile, Santiago, Region Metropolitana, Chile; 6 CARMiG, UTHealth and Center for Infectious Diseases, UTHealth School of Public Health, Houston, TX; Molecular Genetics and Antimicrobial Resistance Unit and International Center for Microbial Genomics, Universidad El Bosque, BOG, COL, Houston, Texas

## Abstract

**Background:**

The global spread of methicillin-resistant *Staphylococcus aureus* (MRSA) is associated with distinct genetic lineages that predominate in specific geographical regions. Available evidence suggests the Chilean-Cordobes clone (ChC), an ST5-SCC*mec*I lineage, has largely predominated in Chilean hospitals since its first description in the late 1990’s. Although the circulation of other MRSA lineages, including community-associated clones, has been well documented, the dynamics of clonal replacement over time has not been explored. Therefore, we aimed to study the molecular epidemiology and dynamics of clonal replacement using a large collection of clinical MRSA strains recovered from Chile during the last two decades.

**Methods:**

We used whole-genome sequencing (WGS) and core-based phylogenomic analysis to identify genetic lineages and explore their relationship in 798 MRSA isolates obtained between 1999-2018 from two tertiary-care Chilean hospitals.

**Results:**

Overall, the most frequently identified clones were the ST5-SCC*mec*I ChC (n=476, 60%), followed by ST105-SCC*mec*II (n=119, 15%), ST72-SCC*mec*IV (n=74, 9%), and ST8-SCC*mec*II (n=26, 3%). Phylogenomic reconstruction demonstrated 7 major clades: Clade I (CC30); Clade II (CC22); Clade III (CC97); Clade IV (CC8); Clade V (ST72); Clade VI (CC5/ST225 and ST105) and Clade VII (CC5/ST5-SCC*mec*I) (Fig. 1). The ChC clone remained the most frequent MRSA lineage throughout the study period (Fig. 2). However, its relative abundance decreased from >90% of isolates in 1999 to ca. 40% in 2018. This decrease began around 2005 and was associated with a progressive expansion of the ST105-SCC*mec*II and ST72-SCC*mec*IV lineages (Fig. 2). A Bayesian molecular clock analysis established the most recent common ancestor in 1964 (95% HPD interval=1961.975-1966.218) and corroborated a CC5 expansion event starting in Chile in 1999 (Fig. 3). Interestingly, our analyses revealed two branches within the ST5-SCC*mec*I lineage: one predominating in 1999-2006, and a more recent branch (related to the ST105-SCC*mec*II clone) that emerged around 2008.

Figure 1. Core genome phylogenomic reconstruction of the 798 MRSA isolates.

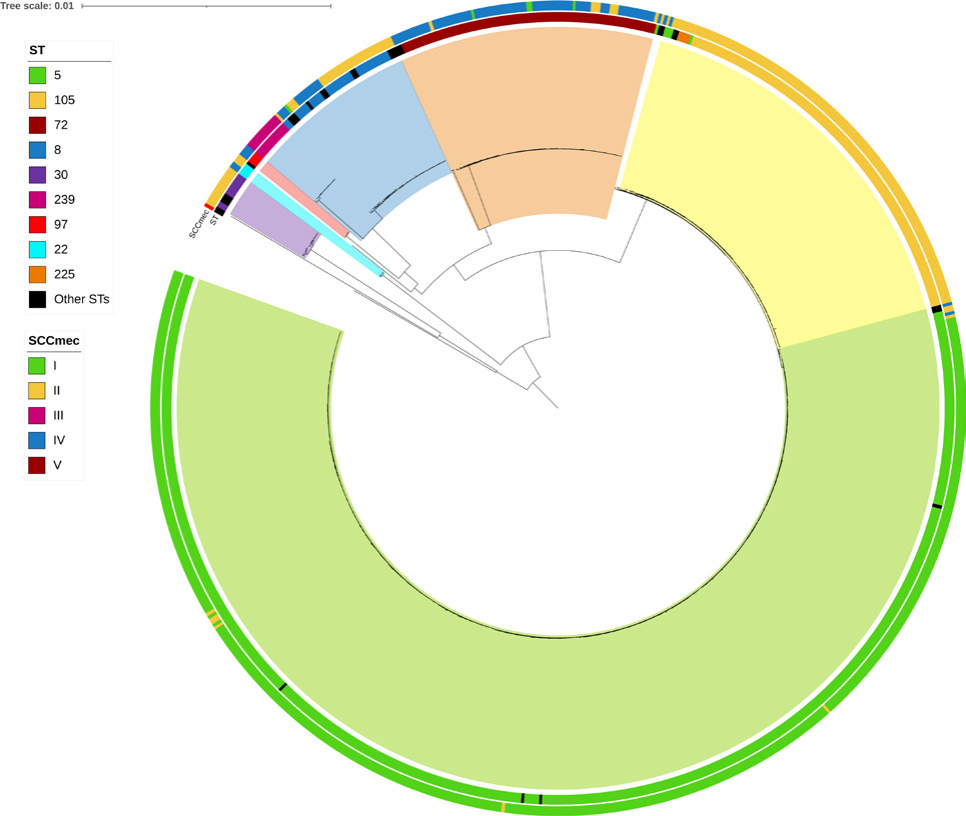

The seven major clades are represented by colored sections. The Clade I (purple section) was composed of isolates belonging to the CC30. Clade II (cyan section) includes four isolates of CC22. Clade III (red section) is composed of isolates of CC97. Clade IV (blue section) grouped isolates of different ST239 and ST8, belonging to the CC8. Clade V (orange section) includes isolates of ST72. Clade VI (yellow section) includes isolates of ST225 and ST105, both belonging to CC5. Clade VII (green section) is mostly composed of isolates of ST5-SCCmecI. The inner ring shows the ST of the isolates; the external ring shows the staphylococcal chromosomal cassette mec (SCCmec) type.

Figure 2. Relative frequency of MRSA clones from 1999 to 2018.

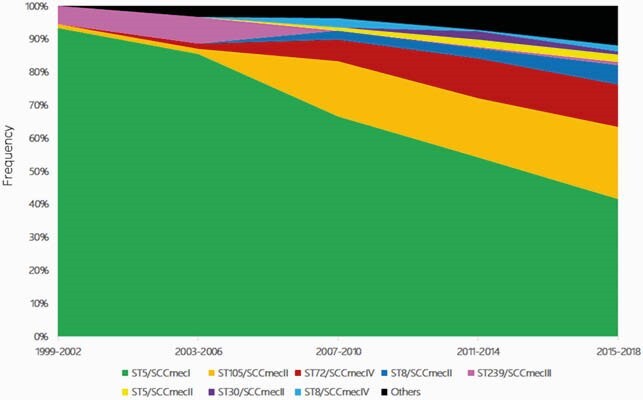

The genomes were grouped according to their isolation dates. Most frequent MRSA clones are represented by colored sections.

Figure 3. Maximum clade credibility tree from the molecular clock analysis of the 798 MRSA genomes.

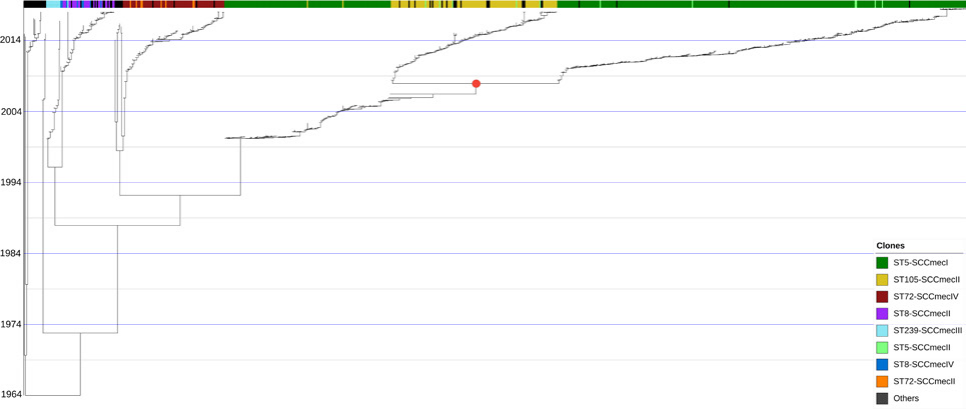

A Bayesian molecular clock analysis was performed with BEAST using the isolation date of each genome as a calibrator. The colored strip showed the most frequent clones. The red dot shows a major event of divergence in 2008.

**Conclusion:**

The ChC clone remains the most prevalent MRSA in Chile. However, our data is consistent with the evolution of this clone and a progressive replacement of with ST105 and ST72 genetic lineages.

**Disclosures:**

**Lorena Diaz, PhD** , Nothing to disclose **Cesar A. Arias, M.D., MSc, Ph.D., FIDSA**, **Entasis Therapeutics** (Grant/Research Support)**MeMed Diagnostics** (Grant/Research Support)**Merk** (Grant/Research Support)

